# Muscle Synergies of the Lower Extremities During Gait Initiation in Individuals With and Without Chronic Ankle Instability

**DOI:** 10.1002/jfa2.70077

**Published:** 2025-08-25

**Authors:** Shaghayegh Zivari, Mohammad Yousefi, Abbas Farjad Pezeshk, Teddy Caderby

**Affiliations:** ^1^ Department of Sport Biomechanics Faculty of Sport Sciences University of Birjand Birjand Iran; ^2^ Laboratoire IRISSE—EA 4075 UFR des Sciences de l’Homme et de l’Environnement Université de La Réunion, Le Tampon La Réunion France

**Keywords:** chronic ankle instability, gait initiation, muscle synergies

## Abstract

**Background:**

Chronic ankle instability (CAI) disrupts postural stability after ankle sprains and inadequate treatment. Gait initiation (GI), governed by central nervous system (CNS) patterns, is used to evaluate stability. Muscle synergy, which reflects coordinated activations, reveals neuromuscular control. This study investigates lower limb muscle synergies during GI in individuals with and without CAI to understand their neuromuscular strategies.

**Design:**

Cross‐sectional study.

**Setting:**

Laboratory.

**Method:**

This study involved 20 participants, 10 healthy men and 10 patients with CAI. Six electrodes were applied per the SENIAM guidelines, and markers were set according to the cluster model. The participants initiated gait after an auditory cue was presented on a force plate. OpenSim simulated a musculoskeletal model using kinematic and muscle activity data. Muscle synergies were analyzed via HALS in MATLAB. Statistical tests, including Wilcoxon and one‐way ANOVA, were conducted in SPSS with *p* < 0.05 as the significance threshold.

**Results:**

The number of muscle synergies was not significantly different between the healthy and CAI groups (*p* > 0.05). However, muscle weight differed significantly between synergies 1 and 2 (*p* < 0.05). In synergy 1, the TA had greater weighting in the CAI group, whereas synergy 2 had higher RF and GM_L weightings in the CAI group. Synergy 3 revealed greater PL weight in the control group (*p* < 0.05).

**Conclusion:**

In CAI, PL muscle weakness is offset by the TA, RF, and GM_L muscles resulting in altered ankle strategies during gait instability. This compensation disrupts motor chains, increases movement complexity, and involves the CNS, framing CAI as a global movement issue rather than a localized problem.

**Abbreviations**
APA: anticipatory postural adjustments
CAI: chronic ankle instability
CNS: central nervous system
COM: center of mass
CON: control
COP: center of pressure
GC: gastrocnemius
GI: gait initiation
GM: gluteus maximus
Gmed: gluteus medius
PL: peroneus longus
RF: rectus femoris
SL: soleus
TA: tibialis anterior

## 1 Introduction

Lateral ankle sprain remains one of the most frequently encountered musculoskeletal injuries in sports and in physically demanding activities [1]. This condition poses a substantial clinical concern as up to 74% of those affected continue to experience residual symptoms—most commonly pain during activity—and a considerable portion eventually develop chronic ankle instability (CAI) [1, 2]. Typically arising after one or multiple acute sprains, CAI is characterized by a range of sensorimotor dysfunctions [3]. These include compromised proprioception, impaired postural control, neuromuscular incoordination, and reduced muscle strength [4]. Such instability not only restricts an individual's capacity to perform daily tasks but also leads to a diminished quality of life when compared to their healthy counterparts [4, 5]. Despite the high prevalence of CAI, its treatment remains contentious, and the development of more effective interventions necessitates a deeper understanding of its underlying neurophysiological mechanisms [2]. Research to date has shown that individuals with CAI exhibit altered neuromuscular control during motor tasks—especially those requiring balance and anticipatory postural regulation [6].

Gait initiation (GI) serves as a particularly insightful task in this context, as it reliably provokes distinct patterns of neuromuscular activity in both healthy and clinical populations [7]. As a transitional movement that shifts the body from a static to a dynamic state, GI demands precise inter‐joint and inter‐limb coordination [8]. Due to its inherent postural destabilization prior to forward motion, GI is recognized as a valuable method for probing postural control mechanisms [9]. Given that instability in posture is central to CAI, examining muscle activation patterns during GI can provide critical insights into the neuromuscular coordination deficits associated with this condition. However, studies examining muscle activity during GI in individuals with CAI have produced inconsistent results [10]. Although some report heightened muscle activation [11], others have found reduced activity [6, 11, 12]. For instance, the peroneus longus has been noted to activate earlier in those with CAI compared to healthy subjects, possibly indicating a compensatory response to lateral ankle instability [13, 14]. Such differences are thought to reflect diminished corticomotor excitability linked to neuromuscular impairments. Under normal circumstances, the peroneus longus contributes to foot eversion and plantarflexion during gait [10]. Interestingly, some studies have also observed increased activation of the tibialis anterior in patients with CAI during the stance phase of walking [3, 15]. Yet, findings from GI tasks are less consistent; in one study, tibialis anterior activity was reduced, resulting in exaggerated plantarflexion prior to heel strike [16]. Furthermore, elevated activation of the gluteus medius and evidence of peroneal dysfunction have also been observed in individuals with CAI [15]. On a biomechanical level, efficient coordination of ankle musculature is essential for generating forward momentum, whereas hip flexors, such as the rectus femoris, are integral to initiating the stepping process [7]. These control demands become even more critical during GI, due to the postural challenges associated with transitioning from stable to dynamic states. Studies of rhythmic motor behaviors, such as walking, suggest that the central nervous system (CNS) orchestrates movement using muscle synergy modules. The stability and effectiveness of locomotor control appear to depend on the consistency and organization of these synergies [17]. Substantial evidence supports the notion that the CNS simplifies movement control by grouping muscles into functional units—synergies—thus avoiding the need to manage each muscle individually [18].

Muscle synergies represent the coordinated activation of multiple muscles, allowing for streamlined control over complex movements by focusing on a limited number of key activation patterns. These patterns help the neuromotor system to handle the body's numerous degrees of freedom efficiently [19]. Because most motor tasks involve coordinated movements across several body segments, any breakdown in intersegmental coordination can interfere with performance in everyday activities. Such disruptions are particularly evident in individuals with altered movement patterns due to injury or disease [20]. In this light, GI—characterized by sudden postural shifts and anticipatory muscle coordination—serves as an effective model for analyzing how CAI might alter motor control strategies.

Muscle synergy analysis has emerged as a robust tool for quantifying coordination and exploring synchronized muscle activity [21]. Two primary types are typically studied: spatial synergies, which involve fixed muscle weightings modulated by varying timing profiles, and temporal synergies, which use consistent timing profiles to coordinate different muscle weightings. A common technique for extracting these synergies is nonnegative matrix factorization (NMF). More recently, the hierarchical alternating least squares (HALS) algorithm has been introduced, offering improved computational speed by continuously refining variables based on the most recent estimates [22]. These analytical methods provide meaningful insights into CNS control strategies, such as the complexity of muscle activation patterns—often indicated by the number of synergies required to perform a task [23]. For example, Kim et al. demonstrated that two to four synergies were sufficient for reconstructing EMG data during each trial, with three being optimal in most cases [24]. Conversely, research by Kibushi et al. suggested that four to five synergies were observed during walking, each aligning with specific gait phases, such as loading response or propulsion [21]. A reduced number of synergies may indicate simplified motor control, a pattern observed in patients with neurological impairments who often exhibit merged or rudimentary synergy patterns compared to healthy individuals. Stroke patients, for instance, have shown to rely on fewer muscle synergies when coordinating lower limb movements during walking [23].

As CAI may affect centrally mediated neuromuscular control similar to other neurological disorders, investigating synergy patterns during GI could offer valuable insights into potential alterations in motor control [24]. Although modified synergy structures have been well‐documented in conditions, such as stroke and cerebral palsy, limited research exists on how CAI might influence such patterns during functional tasks such as GI. This represents a noteworthy gap in current literature [23]. Thus, the aim of this study is to explore and contrast muscle synergies during GI in individuals with CAI versus healthy controls (CONs) using the HALS algorithm. It is hypothesized that those with CAI will exhibit altered synergy configurations and distinct muscle weightings within each synergy, reflecting disruptions in neuromuscular coordination.

## 2 Materials and Methods

### 2.1 Sample Size

To determine the appropriate sample size, calculations were carried out using G*Power version 3.1.9.2. Assuming a medium effect size (Cohen's *d* = 0.5), a significance threshold of 0.05 and aiming for a statistical power of 0.8, the total number of required participants was estimated to be 20. This resulted in two equal groups of 10 individuals. The calculated critical *t*‐value was 1.99 with 66 degrees of freedom, producing an actual statistical power of 0.81.

### 2.2 Participants

Twenty healthy male volunteers participated in this study. They were assigned to one of two groups: the chronic ankle instability (CAI) group and a healthy control (CON) group, each comprising 10 individuals. Demographic characteristics for the CAI group were average age 22.30 ± 2.97 years, height 175.60 ± 7.23 cm, and weight 66.80 ± 9.51 kg. For the CON group, the respective averages were 23.00 ± 1.68 years, 177.45 ± 6.26 cm in height, and 70.45 ± 6.93 kg in weight. All participants were male to minimize variability related to sex‐based differences in neuromuscular behavior, particularly muscle synergy patterns. Although this decision helped improve consistency within the sample, it does restrict how well the findings might generalize to female populations.

Inclusion criteria for the CAI group involved scoring below 24 on the Cumberland Ankle Instability Tool (CAIT) and scoring less than 90% on the daily living subscale and below 80% on the sports subscale of the Foot and Ankle Ability Measure (FAAM) [25]. Additionally, eligible individuals reported at least two previous lateral ankle sprains, with the first occurring at least 12 months prior to the study. Participants with a history of lower limb surgery or fractures were excluded as were those unwilling or unable to provide informed consent. Written informed consent was obtained from all participants before testing began.

### 2.3 Data Collection

Surface electromyography (EMG) was used to record muscle activity, with electrode placement guided by SENIAM recommendations [26]. Prior to placement, each site was shaved and cleaned with isopropyl alcohol. A total of six Ag/AgCl surface electrodes were applied on the dominant limb to target the following muscles: tibialis anterior (TA), soleus (SL), peroneus longus (PL), gluteus medius (Gmed), rectus femoris (RF), and gastrocnemius (GC). The electrodes were placed with a 30‐mm interelectrode spacing, aligned parallel to the muscle fibers. For participants in the CAI group, data were collected from the symptomatic side regardless of dominance. Dominant limb identification was based on the preferred leg used to kick a ball [27].

**FIGURE 1:**
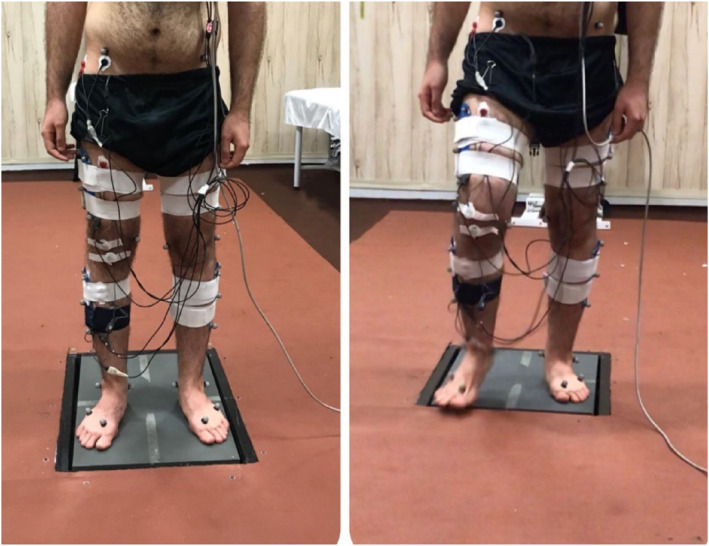
Perform the test. Left: static test and right: gait initiation test.

Before starting the trials, height and weight were recorded, followed by a 15‐min walking warm‐up. For the static test, participants stood barefoot on a force plate in a natural posture for a duration of 10 s. For static trials, participants stood barefoot on a force plate in a relaxed upright position for 10 s, with both feet side‐by‐side and weight evenly distributed. The trial was repeated if the vertical ground reaction force (vGRF) between the legs differed by more than 5% or if visual asymmetries were noticed. During dynamic trials, participants began walking at their self‐selected walking speed following an auditory cue and continued along the walkway (Figure 1). Self‐selected walking speed defined as the natural pace they would typically use during everyday walking—not deliberately fast or slow. Up to three trials were collected per person. Any trial in which more than three motion markers were detached, EMG signal was lost, or excessive noise appeared in the force plate data were excluded.

### 2.4 Data Processing

**FIGURE 2:**
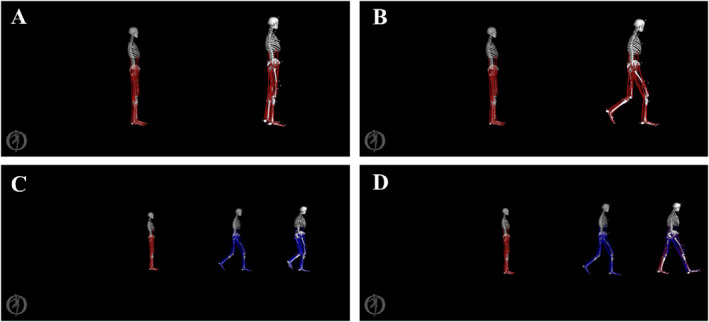
Stages of simulation. (A) = scale: accurate matching of laboratory markers to model markers, (B) = inverse kinematics (IK): finding the angles of each joint, (C) = reduce residual (RRA): finding the torque of each joint, and (D) = computed muscle control (CMC): calculating muscle force separately.

Data collection was performed using a synchronized system involving Vicon motion capture, a Bertec force plate, and a Megavin EMG setup. The motion capture and EMG systems were sampled at 100 Hz. The ground reaction force (GRF) and motion capture data were filtered via a fourth‐order zero‐lag Butterworth filter. EMG data underwent filtering using a fourth‐order zero‐lag Butterworth filter with a 10–500 Hz bandpass, then normalized to peak activation per trial [28]. Muscle force estimations were conducted in OpenSim through a four‐step musculoskeletal model simulation (Figure 2). Finally, computed muscle control (CMC) was determined for each participant.

Computed muscle control (CMC) algorithms provided estimates of muscle force across 92 muscles. However, only the data from the start of gait until the point at which the force plate registered zero were used for further analysis. Thirteen specific muscles were selected for detailed examination: peroneus longus (PL), medial gastrocnemius (MG), soleus (SL), rectus femoris (RF), vastus medialis (VM), tibialis anterior (TA), biceps femoris (BF), semitendinosus (ST), tensor fasciae latae (TFL), gluteus maximus (GM), gluteus medius (Gmed), as well as the left‐side counterparts of gluteus medius (Gmed_L), and gluteus maximus (GM_L). Each activation value was standardized by dividing it by its standard deviation to achieve a unit variance.

Muscle synergy patterns were extracted using MATLAB software via a decomposition model. In this model, **M** refers to the number of synergies, **W** denotes the synergy weight matrix, and **C** represents the temporal activation matrix:

(1) $$M=\sum_{i=1}^{N_{\text{synergy}}}\left(W_{i}C_{i}\right)+\epsilon .$$

The number of muscle synergies was calculated by the variance accounted for (VAF) (Equation 2: ρ is the number of muscles and n is time)

(2) $$\text{VAF}=1-\frac{\sum_{i=1}^{\rho }\sum_{j=1}^{n}{\left(\varepsilon_{i,j}\right)}^{2}}{\sum_{i=1}^{\rho }\sum_{j=1}^{n}{\left(E_{i,j}\right)}^{2}}.$$

The acceptable number of muscle synergies was where the VAF > 90% [29].

### 2.5 Statical Analysis

All statistical analyses were conducted in SPSS version 26. The Shapiro–Wilk test was used to verify data normality. Differences in the number of synergies between the CAI and CON groups were examined using the Wilcoxon rank‐sum test. One‐way ANOVAs were applied to assess between‐group differences in demographic variables. Additionally, an independent *t*‐test was used to assess significant differences in walking speed between the CAI and CON groups. A significance threshold of *p* < 0.05 was used for all comparisons.

## 3 Results

Participants exhibited a self‐selected walking speed at gait initiation. No significant difference was found in this parameter between the CAI and CON groups (Table 1).

**TABLE 1:** Results of independent *t*‐test for walking speed at gait initiation.

Group	Mean (SD)	*p*
CAI	0.51 (0.03)	0.47
CON	0.53 (0.05)	

Abbreviations: CAI, chronic ankle instability; CON, control.

Gait initiation (GI) was divided into three major phases: anticipatory postural adjustment (APA), mid‐swing, and initial double support.

- *APA Phase*: This phase began with the auditory cue and concluded when the center of pressure (COP) shifted toward the stance limb.
- *Mid‐Swing Phase*: Started at heel‐off of the swing leg and ended with its heel contacting the ground. The center of mass (COM) moved toward the stance limb during this phase. Due to notable shifts in muscle activity timing, this phase was further subdivided into mid‐swing 1 and mid‐swing 2.
- *Initial Double Support Phase*: Started when the swing limb's heel contacted the ground and ended at toe‐off of the stance limb. At this point, the COM transitioned fully to the swing limb and the force plate reading dropped to zero.

**FIGURE 3:**
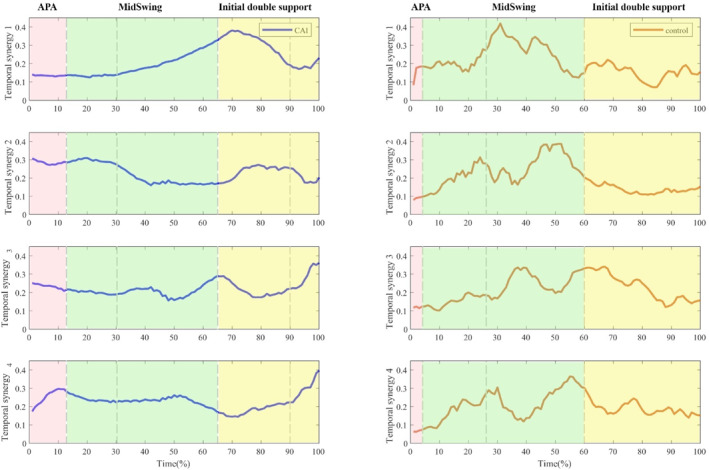
The activation coefficients in CAI and CON groups during GI. GI was divided into anticipatory postural adjustment (APA), mid‐swing, and initial double support, with mid‐swing further separated into mid‐swing 1 and mid‐swing 2.

According to muscle synergy graphs, both groups exhibited the most significant muscle activity during the mid‐swing phase (Figure 3).

The results related to Synergy 1 indicate that individuals with CAI predominantly exhibit muscle activity during the primary initial double support phase (68%–100% of the GI cycle), whereas the CON group engages during the APA phase (0%–10% of the GI cycle). Notably, there was a significant difference in the activation of the tibialis anterior (TA) muscle between the CON group and the CAI group (*p* < 0.05), with the CAI group demonstrating greater involvement of this muscle than the CON group did.

Within Synergy 2, individuals with CAI predominantly exhibit muscle activity during the mid‐swing 1 phase (15%–30% of the GI cycle), whereas the CON group is active during the mid‐swing 2 phase (30%–60% of the GI cycle). Importantly, a significant difference was observed in the activation of the RF and GM_L muscles between the two groups, with the CAI group demonstrating greater engagement of these muscles than the CON group did.

Within Synergy 3, individuals diagnosed with CAI predominantly exhibit muscle activity during the mid‐swing 2 phase (30%–68% of the GI cycle), whereas the CON group is active during the initial double support phase (60%–85% of the GI cycle). This synergy highlights a significant difference in the activation of the PL muscle between the two groups, with the CON group showing a greater contribution from this muscle than the CAI group.

Within Synergy 4, individuals with CAI are primarily active during the anticipatory postural adjustment (APA) phase (0%–15% of the GI cycle), whereas the CON group is engaged in the mid‐swing 1 phase (10%–30% of the GI cycle). Importantly, no significant differences in muscle weight were detected between the CAI and CON groups.

Each synergy was interpreted based on the muscles with the highest weighting coefficients, which were assumed to be the main contributors to that module. This method aligns with prior work in muscle synergy analysis [23].

### 3.1 Dimensionality of Muscle Synergies

**FIGURE 4:**
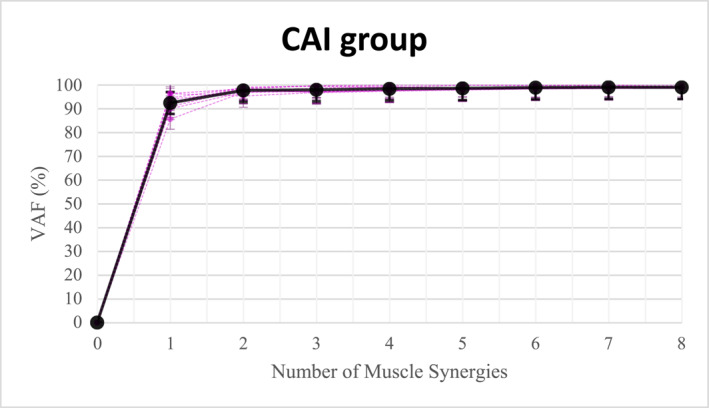
The number of muscle synergies in the CAI group.

**FIGURE 5:**
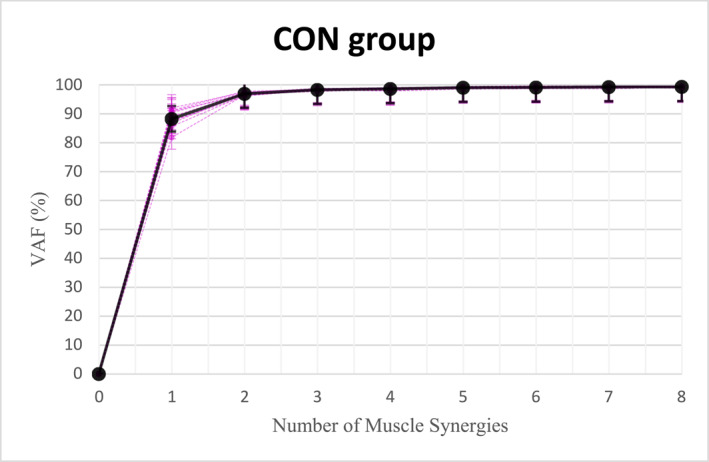
The number of muscle synergies in the control group.

On the basis of the results of the variance accounted for (VAF), two to five synergies were identified as the appropriate number of synergies to represent the EMG data in both the chronic ankle instability (CAI) group (Figure 4) and the control group (CON) (Figure 5). In most cases, four synergies were sufficient to reconstruct the EMG data. According to the Wilcoxon rank‐sum test, there was no significant difference in the number of muscle synergies between the CAI and CON groups during gait initiation (*p* = 0.67, which was greater than 0.05) (Table 2).

**TABLE 2:** Results of the Wilcoxon rank‐sum test.

Group	*Z*	*p*
CAI and CON	−0.42	0.67

Abbreviations: CAI, chronic ankle instability; CON, control.

### 3.2 Functional Interpretation of Muscle Synergies

**FIGURE 6:**
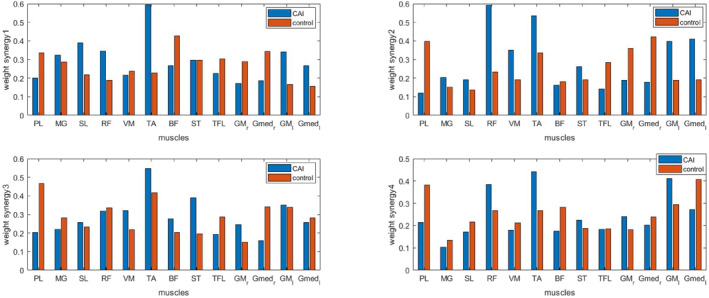
Muscle weighting within each synergy in both groups.

**FIGURE 7:**
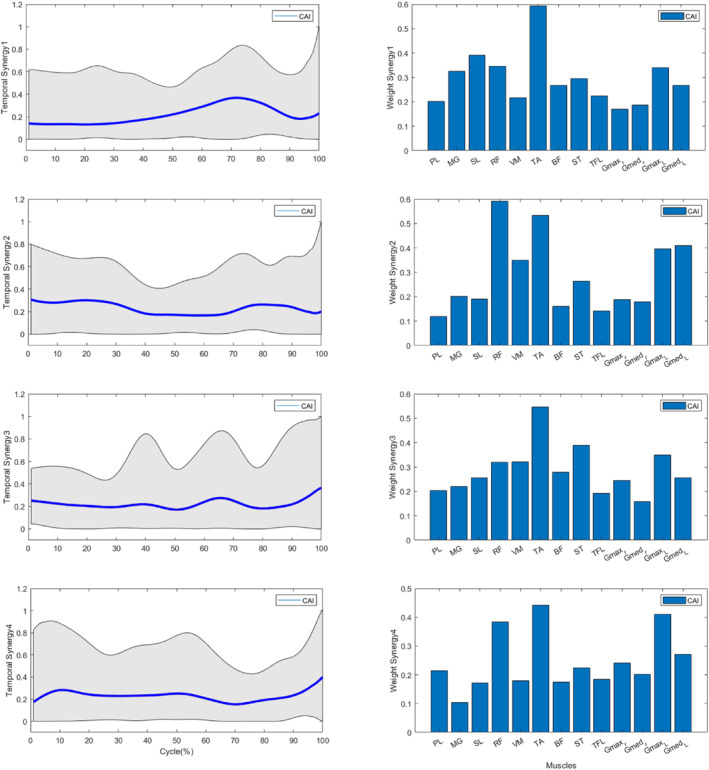
Temporally weighted data for the 4 synergies in the CAI group.

**FIGURE 8:**
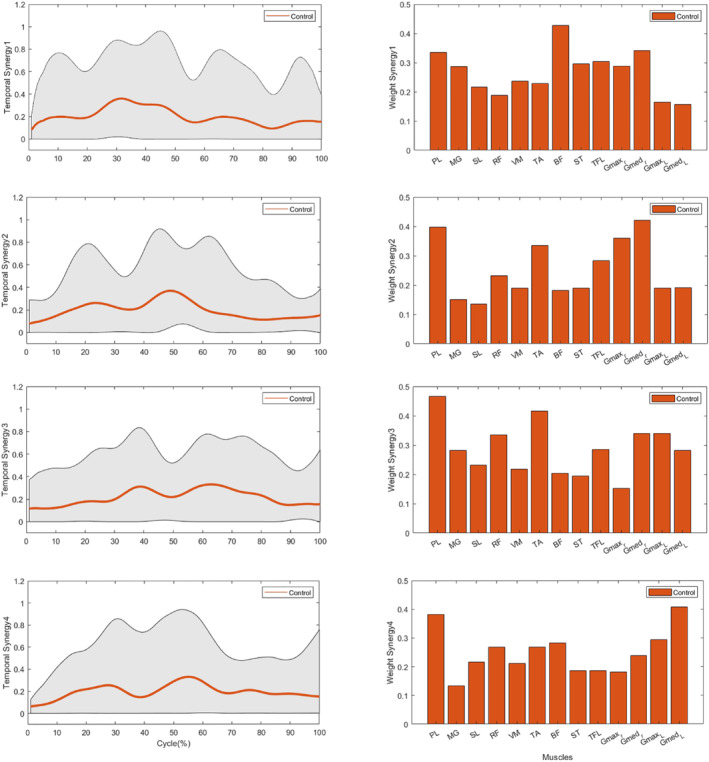
Temporally weighted data for the 4 synergies in the control group.

The analysis of muscle weightings within each synergy was conducted for both groups (Figure 6). Significant differences were observed in the muscle weights of the TA within the synergy vector of Synergy 1 (*p* < 0.05), with the weights of the TA being greater in the CAI group than in the control group (Table 3). Additionally, significant differences in muscle weight were found for RF and GM_L within the synergy vector of Synergy 2 (*p* < 0.05), indicating that the weightings of these muscles were greater in the CAI group than in the control group (Table 4). Additionally, significant differences were observed in the muscle weight of the PL within the synergy vector of Synergy 3 (*p* < 0.05), with the weight of the PL muscle being greater in the control group than in the CAI group (Table 5). No significant differences were found in the muscle weightings within Synergy 4, and no differences were noted in the weightings of other muscles across all synergies (Table 6). Temporal weightings for each synergy were presented for both the CAI (Figure 7) and CON groups (Figure 8).

**TABLE 3:** Results of ANOVA within synergy 1.

Variable	Group	Mean (SD)	*p*
TA	CAI	0.59 (0.19)	*0.001
	CON	0.22 (0.25)	
RF	CAI	0.34 (0.40)	0.25
	CON	0.18 (0.12)	
GM_L	CAI	0.34 (0.21)	0.10
	CON	0.16 (0.24)	
PL	CAI	0.20 (0.33)	0.30
	CON	0.33 (0.22)	

Abbreviations: CAI, chronic ankle instability; CON, control; GM_L, gluteus maximus left; PL, peroneus longus; RF, rectus femoris; TA, tibialis anterior.

^*^ Indicates a significant difference (*p* < 0.05).

**TABLE 4:** Results of ANOVA within synergy 2.

Variable	Group	Mean (SD)	*p*
TA	CAI	0.53 (0.19)	0.15
	CON	0.33 (0.37)	
RF	CAI	0.59 (0.37)	*0.01
	CON	0.23 (0.22)	
GM_L	CAI	0.39 (0.18)	*0.02
	CON	0.18 (0.18)	
PL	CAI	0.11 (0.21)	0.07
	CON	0.39 (0.41)	

Abbreviations: CAI, chronic ankle instability; CON, control; GM_L, gluteus maximus left; PL, peroneus longus; RF, rectus femoris; TA, tibialis anterior.

^*^ Indicates a significant difference (*p* < 0.05).

**TABLE 5:** Results of ANOVA for synergies 3.

Variable	Group	Mean (SD)	*p*
TA	CAI	0.54 (0.21)	0.33
	CON	0.41 (0.35)	
RF	CAI	0.31 (0.28)	0.88
	CON	0.33 (0.21)	
GM_L	CAI	0.34 (0.18)	0.93
	CON	0.33 (0.34)	
PL	CAI	0.20 (0.26)	*0.03
	CON	0.46 (0.25)	

Abbreviations: CAI, chronic ankle instability; CON, control; GM_L, gluteus maximus left; PL, peroneus longus; RF, rectus femoris; TA, tibialis anterior.

^*^ Indicates a significant difference (*p* < 0.05).

**TABLE 6:** Results of ANOVA within synergy group 4.

Variable	Group	Mean (SD)	*p*
TA	CAI	0.44 (0.22)	0.33
	CON	0.21 (0.24)	
RF	CAI	0.38 (0.35)	0.10
	CON	0.26 (0.30)	
GM_L	CAI	0.41 (0.13)	0.31
	CON	0.29 (0.32)	
PL	CAI	0.21 (0.26)	0.19
	CON	0.38 (0.28)	

Abbreviations: CAI, chronic ankle instability; CON, control; GM_L, gluteus maximus left; PL, peroneus longus; RF, rectus femoris; TA, tibialis anterior.

## 4 Discussion

This study aimed to investigate lower extremity muscle synergy during gait initiation (GI) in individuals with and without chronic ankle instability (CAI). The findings provide novel insights that contribute to the foundational understanding of muscle synergy in individuals with CAI, offering a basis for future research. The results revealed no significant differences in the number of muscle synergies during GI between CAI and control (CON) groups. Based on muscle synergy analysis, four synergies were identified as appropriate for participants in both groups. This observation contradicts the initial hypothesis, which anticipated differences in the number of synergies in individuals with CAI. However, individuals with CAI exhibited distinct muscle weightings, with increased tibialis anterior (TA) weighting in Synergy 1, and greater rectus femoris (RF) and lateral gastrocnemius (GM_L) weightings in Synergy 2, alongside reduced peroneus longus (PL) weighting in Synergy 3 compared to the CON group. These findings support the hypothesis that individuals with CAI exhibit altered weight‐related muscle synergies, reflecting distinct neuromuscular control mechanisms during GI.

### 4.1 Number of Muscle Synergies

No significant differences in the number of muscle synergies were observed between the groups. Both groups displayed a synergy count of four, meeting the ≥ 90% variance accounted for (VAF) criterion, consistent with prior research by Kim et al. (2021), who found comparable synergy counts across groups [24]. Although literature reports varying synergy ranges—for instance, Mehrabi et al. (2019) identified 4–6 synergies in healthy individuals [30] and Kiboshi et al. reported 4–5 synergies during walking [21]—our findings align with studies indicating that individuals with neurological impairments, such as stroke, may exhibit fewer synergies, potentially reflecting simpler cerebral processing requirements [23]. Despite initial expectations of reduced synergy counts in CAI, this was not supported by the data demonstrating comparable synergy numbers in both groups.

### 4.2 Weightings of Muscle Synergies

Research on muscle activity underscores the distinct activation patterns of various muscles across different phases of the GI cycle [31]. The primary mechanism facilitating the posterior shift of the center of pressure (COP) involves bilateral inhibition of tonic gastrocnemius/soleus (SL) activity, followed by a bilateral phasic burst of tibialis anterior (TA) activation [32]. This interaction generates an external dorsiflexion moment at the ankles, enabling forward body rotation over the feet. During the stance phase of gait, hip abductors play a crucial role in controlling the frontal plane motion of the center of mass (COM) and in managing the lateral loading and unloading mechanism. Both hip adductors and abductors are integral to this process during GI [32]. In typical walking patterns, hamstrings primarily contribute to backward force generation during the initial heel strike but exhibit diminished activity throughout most of the stance phase [31]. Other hip extensors, particularly the gluteus maximus (GM), assist in generating backward force during the loading response phase, working synergistically with TA. This interplay between hip extensors and ankle dorsiflexors during the early stance phase helps decelerate forward leg motion [31]. Mid‐stance is characterized by the engagement of ankle plantar flexors, including SL and gastrocnemius, to facilitate forward movement. The rectus femoris (RF) supports forward progression, albeit to a lesser extent than the iliopsoas [32]. The peroneus longus (PL) is pivotal in propulsion by maintaining lateral dynamic ankle stability during weight‐bearing [33] and is particularly crucial during both heel‐off and subsequent heel‐strike phases [34].

The study findings revealed notable differences in muscle weight distribution across synergy combinations between individuals with CAI and those in the CON group. Specifically, Synergy 3 demonstrated reduced PL weighting in the CAI group, suggesting possible impairments in PL function. The PL muscle serves a critical protective function, mitigating inversion ankle sprains [32, 35], especially during single‐limb stance phases where such sprains are most prevalent [36]. The PL also plays a vital role in regulating supination [34], and its dysfunction has been widely associated with ankle instability [37]. Prior research highlights PL muscle weakness as a key factor in CAI [34, 35, 38], with Coldenhoven reporting diminished PL activation ranges in CAI individuals, which may heighten their susceptibility to ankle sprains [38]. Although Santilli et al. observed decreased peroneal activation in the injured limb [34], Feger reported increased PL neuromuscular activity within the first 80 milliseconds postheel strike in patients with CAI [33]. This heightened activation likely reflects compensatory efforts to maintain frontal plane stability during gait [23]. Moreover, peroneal activation typically intensifies during single‐limb support or when navigating unstable movements underscoring its stabilizing function [39].

Importantly, lateral ankle sprains frequently occur during high‐demand activities, such as jumping or landing in sports. However, repeated sprain episodes may lead to chronic instability, characterized by neuromuscular alterations extending beyond the initial injury. Although GI itself does not directly contribute to injury mechanisms, it provides a relevant context to observe altered muscle coordination patterns caused by instability. Individuals with CAI may exhibit compromised neuromuscular control even during relatively low‐demand tasks, such as walking or GI. This study emphasizes the impact of instability on motor control rather than suggesting GI as a causal factor for ankle sprains.

Our findings showed increased TA weighting in Synergy 1 among individuals with CAI. Various studies have examined the functional role of the TA muscle. Coldenhoven documented reduced TA activation in patients with CAI, leading to increased COP variability and heightened sprain risk [38]. Conversely, other studies reported elevated TA activity during the anticipatory postural adjustment (APA) phase of GI [13, 14, 15], suggesting a compensatory strategy to restrict anterior COP displacement [13]. Hopkins further identified increased TA activity during the stance phase, interpreting dorsiflexion as a motor strategy to stabilize the ankle [15, 23]. Electromyographic (EMG) analyses additionally highlighted that bilateral SL silencing and heightened ipsilateral TA activity facilitate stance phase knee flexion, whereas RF activation supports hip flexion [40].

The CAI group also demonstrated increased RF and GM_L weightings within Synergy 2. In the CON group, RF is typically activated during initial contact and early stance phases to absorb impact [41]. However, Lin et al. reported reduced RF activation in individuals with CAI, which impairs shock absorption [42]. In contrast, Akbas observed heightened RF activation in CAI participants [41], consistent with our findings. Feger noted earlier RF activation in patients with CAI [33], potentially compensating for reduced pronation [43]—an essential element for shock absorption and mid‐stance support [33]. Similarly, earlier GM_L activation after heel contact was identified in individuals with CAI [42], aligning with our findings. The hamstrings, integral to the support moment during normal gait [31], play a diminished role in individuals with CAI as indicated by studies reporting varied findings regarding GM activation [14, 43].

Nonetheless, our results suggest that patients with CAI employ compensatory neuromuscular patterns [33] involving increased activation of RF and GM_L to counteract deficiencies in lateral stability and dynamic ankle support. These findings highlight a neuromuscular compensatory strategy in individuals with CAI, often referred to as the “ankle strategy.” This involves sequential TA activation for dorsiflexion, RF and GM_L engagement to maintain knee extension, and facilitation of center‐of‐mass progression. Weakness in PL, as seen in Synergy 3, likely redistributes functional demands to other muscles within the kinetic chain elevating their activity levels.

Although ankle sprains predominantly occur during high‐load activities, the chronic instability they cause significantly impacts neuromuscular coordination even during controlled tasks such as GI. The study confirms that individuals with CAI exhibit altered muscle synergies and redistributed muscle weights, reflecting impaired motor control. By examining these subtle yet impactful deficits, GI provides a valuable lens for understanding CAI‐related neuromuscular dysfunction.

### 4.3 Limitations

This study has several limitations. First, the small sample size (*n* = 20) may limit the generalizability of findings. Second, the analysis was confined to linear GI in a controlled laboratory setting, suggesting the need for future studies to explore multidirectional tasks and real‐world conditions. Third, data collection was restricted to the dominant leg; bilateral analyses could yield additional insights. Finally, the inclusion of only male participants, although enhancing internal consistency, limits the applicability of findings to female populations. Further research should investigate sex‐specific neuromuscular patterns in CAI.

## 5 Conclusion

The study highlights distinct neuromuscular strategies in individuals with CAI, characterized by altered muscle synergy weightings, particularly reduced PL contribution, compensated by increased TA, RF, and GM_L activity. These findings emphasize the role of disrupted CNS coordination in CAI, advocating for comprehensive rehabilitation protocols that address both local musculoskeletal and global neuromuscular deficits. By leveraging insights into muscle synergy alterations, clinicians can design targeted interventions to restore normal motor control and reduce CAI‐related functional impairments.

## Author Contributions

**Shaghayegh Zivari:** data curation, formal analysis, software, writing – original draft, writing – reviewing and editing. **Mohammad Yousefi:** conceptualization, data curation, formal analysis, software, methodology, supervision, project administration, writing – reviewing and editing. **Abbas Farjad Pezeshk:** methodology, writing – reviewing and editing. **Teddy Caderby:** methodology, writing – reviewing and editing.

## Ethics Statement

The participants were first briefed on the purpose of this study. They were assured that their information would be kept confidential and that their involvement was entirely voluntary. Additionally, they were made aware of their right to withdraw from the study at any time. Informed consent was secured from all participants before their participation. This study was approved by the Ethics Committee of Kharazmi University Research Institute (IR.KHUKRC.1000.135).

## Consent

The participant provided written informed consent for her/his personal or clinical details along with any identifying images to be published in this study.

## Conflicts of Interest

The authors declare no conflicts of interest.

## Data Availability

The authors have nothing to report.
